# A Multimodal Hair-Loss Treatment Strategy Using a New Topical Phytoactive Formulation: A Report of Five Cases

**DOI:** 10.1155/2021/6659943

**Published:** 2021-02-04

**Authors:** Sanusi Umar, Marissa J. Carter

**Affiliations:** ^1^Department of Medicine, Dermatology Division, University of California at Los Angeles, Los Angeles, CA, USA; ^2^Division of Dermatology, Harbor-UCLA Medical Center, Torrance, CA, USA; ^3^Dr. U Hair and Skin Clinic, Manhattan Beach, CA, USA; ^4^Strategic Solutions, Inc., Bozeman, MT, USA

## Abstract

*Introduction*. Current approved medications for hair loss, such as topical minoxidil and oral finasteride, may have suboptimal efficacy or side effects precluding continued use in some patients. Thus, we report an evaluation of the efficacy, safety, and tolerability of a new topical botanical formulation -GASHEE containing over 12 phytoactive ingredients that affect multiple targets in the cascade of pathophysiologic events that cause hair loss. Five patients with various hair-loss conditions, including cases of previous treatment failures, are presented. *Case Presentation*. This is a case series of four women and one man with hair loss due to various causes, four of whom had failed minoxidil treatment for over a year. All patients used the topical treatment as a sole therapy for at least 3 months before the documentation of outcomes, which involved interval changes noted through each patient's account, direct observation, and photography. *Discussion*. In all patients, we observed significant improvements in hair regrowth in the nape, crown, vertex, and temple areas after 3–15 months of treatment. All patients were highly satisfied with their results and reported no adverse events. Although the use of botanicals in the treatment of hair loss is in an infant stage, the new formulation used in this study demonstrated a good efficacy related to hair growth, warranting further evaluation.

## 1. Introduction

**Figure 1:**
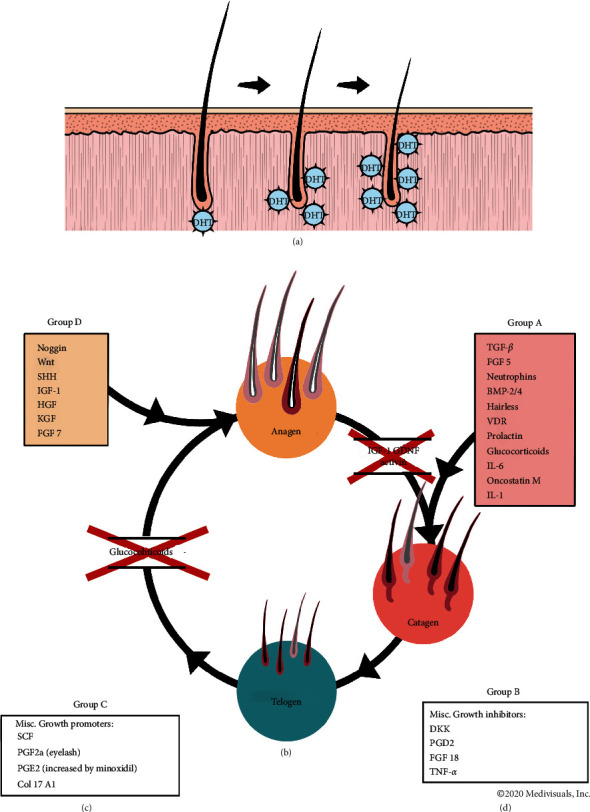
The hair follicle cycle showing the effects of various cytokines, growth promoters, and growth inhibitors regarding different stage transitions. BDNF, brain-derived neurotrophic factor; BMP-2/4, bone morphogenetic protein-2/4; col 17 A1, collagen-type XVII *α* 1 chain; DKK, Dickkopf-related protein; FGF5/7/18, fibroblast growth factor 5/7; GDNF, glial cell line-derived neurotrophic factor; HGF, hepatocyte growth factor; IGF-1, insulin-like growth factor-1; IL-1/6, interleukin-6; KGF, keratinocyte growth factor; PG D2/E2/F2a, prostaglandin D2/E2/F2a; SCF, stem cell factor; SHH, sonic hedgehog; TGF, transforming growth factor; TNF-*α*, tumor necrosis factor *α*; VDR = vitamin D receptor; WNT, wingless-type integration site.

Androgenetic alopecia (AGA, also called male and female pattern alopecia) is the most common cause of hair loss and is estimated to affect 30–58% of men by age 50 and 12–40% of women depending on age and race [1–4]. It is characterized by progressive hair follicular miniaturization caused by the actions of androgens on epithelial cells of genetically susceptible hair follicles in androgen-dependent areas [5]. While complex genetic inheritance and age of the individual are major risk factors in AGA development, on a cellular level, the initiation of an inflammatory condition in the follicle microenvironment is considered the central event, with contributory mechanisms, including abnormal signal transduction (the wingless-type integration site pathway), high levels of apoptosis, and oxidative stress [6–11]. This supports the need for a multifaceted approach for the treatment of AGA, managing central causes, such as 5-dihydrotestosterone (DHT), oxidative stress, and inflammation, as well as downstream factors that precipitate hair loss by adversely affecting hair stem cells and hair cycles (Figure 1). For instance, DHT, a major factor causing hair loss in AGA, has been found to act downstream by inducing the production of interleukin- (IL-) 6 and transforming growth factor- (TGF-) *β*2 by dermal papilla cells (DPCs), thus suppressing hair growth and premature onset of the catagen phase [12, 13].

To date, there is no permanent cure for AGA, which often precipitates anxiety and depression in affected patients [14]. Two drugs, topical minoxidil and oral finasteride, have been approved by the United States Food and Drug Administration for AGA, with finasteride restricted to men because of teratogenicity in women of child-bearing age and poor effects in postmenopausal women. While their mechanism of action is not fully understood, minoxidil is believed to shorten the telogen phase, accelerate the telogen-exogen phase, initiate premature entry of resting hair follicles into the anagen phase, and increase hair follicle size [15]; in contrast, finasteride is a type II 5-*α* reductase that inhibits the final conversion step of testosterone to its active form, DHT [16]. Both drugs can curtail progressive hair loss and stimulate new hair growth. However, their efficacy is suboptimal. Studies have shown that, after 1 year and 4 months of administering oral finasteride and 5% topical minoxidil, respectively, only 48% and 38.6% of the subjects, respectively, experienced hair growth [17, 18]. Additionally, a study revealed that finasteride was ineffective in treating hair loss in postmenopausal women [19]. Furthermore, finasteride is contraindicated in women of childbearing age because of teratogenicity concerns (Propecia (U.S. package insert) 2013 Merck: Whitehouse Station, NJ), while some men would experience sexual dysfunction from its use. Scalp irritation may occur due to topical minoxidil, which, in addition to instances of suboptimal efficacy, has been cited by some patients as a reason for seeking botanical alternatives to pharmaceuticals [20, 21]. Therefore, there is an unsatisfied need for additional safe and effective therapies for AGA.

Another cause of hair loss is traction alopecia (TA), which results from continuous and prolonged tension to the hair. It is most commonly observed in Black/African American women and children who wear hairstyles that pull excessively at the frontotemporal hairline [22]. TA typically affects the frontal and temporal scalp, as well as anterior and superior to the ears. Hairstyles reported by patients with TA include tight braids, weaves, cornrows, ponytails, chignons, or religious head coverings. It should be noted that traction applied to chemically treated hair (i.e., relaxers and dyes) can further increase the risk of TA [23]. The finding of retained hairs along the frontal and/or temporal hairline (“fringe sign”) can help in making a clinical diagnosis of TA [24]. Apart from avoiding all the practices that lead to TA, treatment options have included intralesional corticosteroids directed at the periphery of hair loss to suppress perifollicular inflammation [25], antibiotics used early in the disease for their anti-inflammatory effect [26], minoxidil [27], and hair transplantation.

Central centrifugal cicatricial alopecia (CCCA) is another common cause of alopecia in African American women, perhaps only exceeded in frequency of hair loss by TA, with a likely prevalence of 3–7%, although its true prevalence in various populations is unknown [28]. A genetic basis involving mutations in *PAD13*, which encodes a protein that is essential for proper hair-shaft formation, has been associated with CCCA [29]. Harsh hair grooming practices may precipitate CCCA in genetically predisposed individuals. The scarring alopecia that results from CCCA occurs mainly on the vertex of the scalp with symmetric spread in a centrifugal pattern, hence the name of the disease. The presence of a peripilar white halo as a feature found under dermoscopy, along with other findings, suggests CCCA in an African American patient with mild central thinning. Medical treatments similar to those used for TA have been used, as well as hydroxychloroquine and doxycycline, although all are inadequate and mostly ineffective.

Lastly, there is diffused thinning in postmenopausal women, likely due to the decline in estrogen, which protects hair in women, which would have succumbed to hair loss from androgenic pattern hair loss [30]. Men manifest hair loss from androgenic alopecia early in life because they lack estrogen which protects women from the same problem. However, during and after menopause, the protective effect of estrogen is lost. This accounts for the onset of hair loss, which typically manifests as diffuse hair thinning. Estrogen supplementation is generally not an option because of the inherent dangers of increased risk of breast cancer, as well as other complications of estrogen treatment. As a result, minoxidil is used but is often not effective.

Botanical extracts are obtained from whole plants or specific plant parts, such as the flower, stem, leaves, bark, root, or fruit, and are delivered topically or as dietary supplements. Extracts are typically complex mixtures consisting of numerous individual phytochemical constituents and potential contaminants. Their use as therapeutics has expanded enormously in the last few decades. This approach is attractive, as its wide availability and low cost will allow many different formulations to be devised to address specific disease-related problems. However, a few botanicals are subject to regulation or rigorous research, as they are not labeled for a specific disease [31]. Consequently, their effectiveness or safety is often unclear.

We report the treatment outcomes of five patients treated with a new topical lotion consisting of over 12 phytoactive botanical ingredients and discuss the possible mechanisms of action using published literature.

## 2. Case Presentation

### 2.1. Evaluation of Dr. UGro Gashee® Efficacy and Safety

The study was conducted in accordance with the Declaration of Helsinki (1964). All patients signed informed consent forms.

A diverse group of five patients (in terms of age, sex, race, and cause of hair loss) who exclusively used Dr. UGro Gashee**®** (GASHEE) for their hair treatment is reported. They all reported a high satisfaction level and safety profile after using the topical cosmeceutical.

### 2.2. Case 1

**Figure 2:**
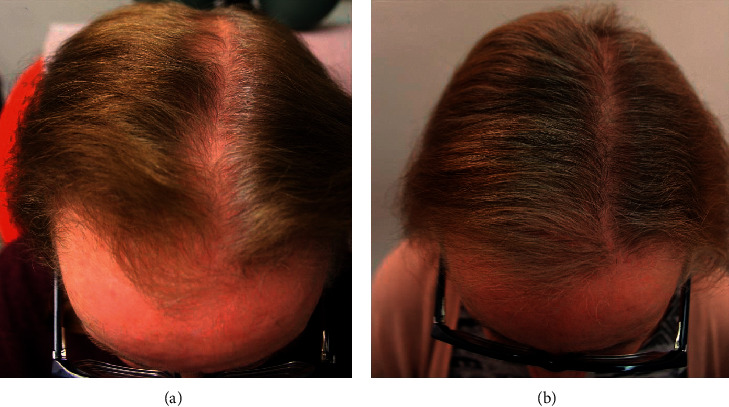
Patient 1: a 68-year-old Caucasian woman. Top of the head before (a) and after (b) 3 months of treatment.

**Figure 3:**
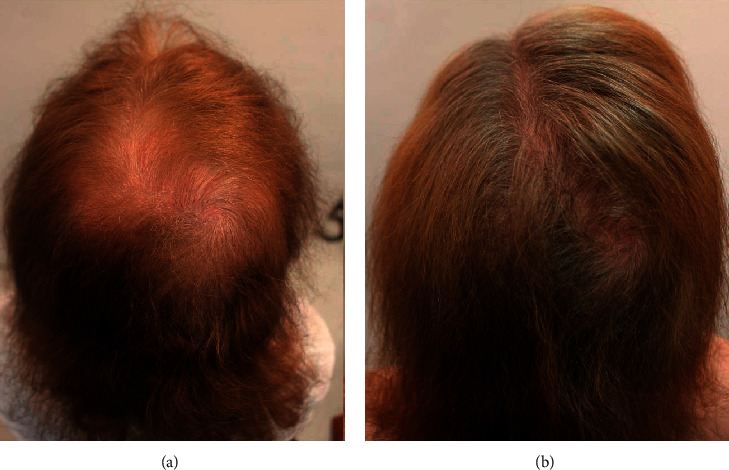
Patient 1: back of the head before (a) and after (b) 3 months of treatment.

**Figure 4:**
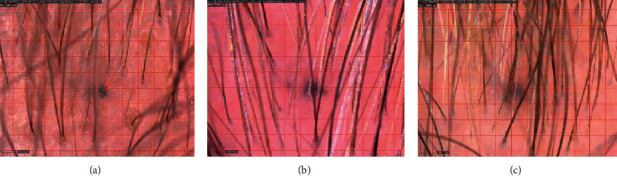
Trichograms of a tattooed spot in the hair path line area of the midscale from patient 1, performed at 2, 3, and 15 months ((a), (b), and (c), respectively). A progressive interval improvement in hair density and caliber was noted.

A 68-year-old Caucasian woman had AGA with significant hairline recession and thinning of the front, top, and crown. She desired to stop her hair loss, as well as stimulate hair growth in the affected areas, and therefore began applying 2% topical minoxidil solution to the affected area as well as taking oral hair- and nail-growth supplements. After over 1 year of using minoxidil without noticeable results, she discontinued both treatments (Figures 2(a) and 3(a)). She began applying the GASHEE lotion formulation to her scalp once at night for 3 months. After leaving the lotion on her scalp throughout the night, she washed her scalp each morning. In between washings, she noted a reduction in the rate of hair shedding. After 3 months, new hair growth in the affected area was observable, and her hair loss completely stopped (Figures 2(b) and 3(b)). Trichograms (Dino-Lite 1.3MP, AnMo Electronics Co., New Taipei City) of a tattooed spot in the hair path line area of the midscale, performed at 2, 3, and 15 months, revealed progressive interval improvement in hair density and caliber (Figures 4(a)–4(c)). She reported no adverse effects.

### 2.3. Case 2

**Figure 5:**
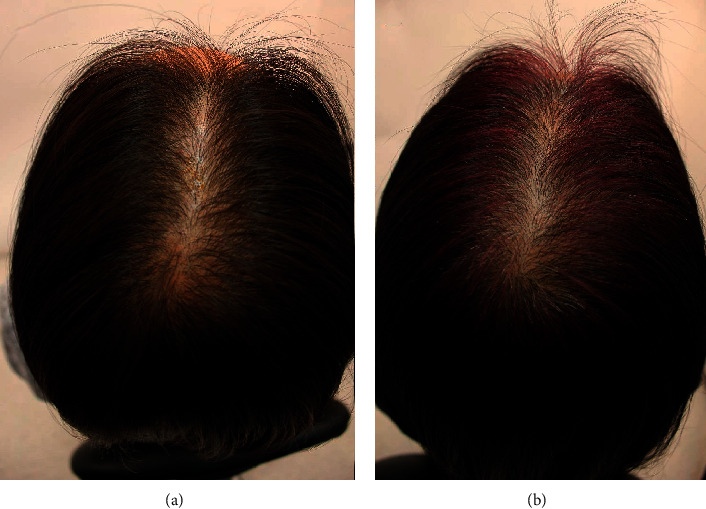
Patient 2: a 48-year-old Chinese woman. Top of the head before (a) and after 3 months of treatment (b).

A 48-year-old, premenopausal, Chinese woman with hair loss caused by female-pattern hair loss (Figure 5(a)) had a family history of advanced female- and male-pattern hair loss, specifically in her mother and brother. She washed her hair three to five times a week and typically kept her hairstyle natural. She did not want to use pharmaceuticals or any oral treatments and began applying GASHEE lotion to her hair and scalp every night. After 3 months of using the product, she reported hair growth in the areas where the spray lotion was applied to her scalp (Figure 5(b)), and no adverse effects were reported.

### 2.4. Case 3

**Figure 6:**
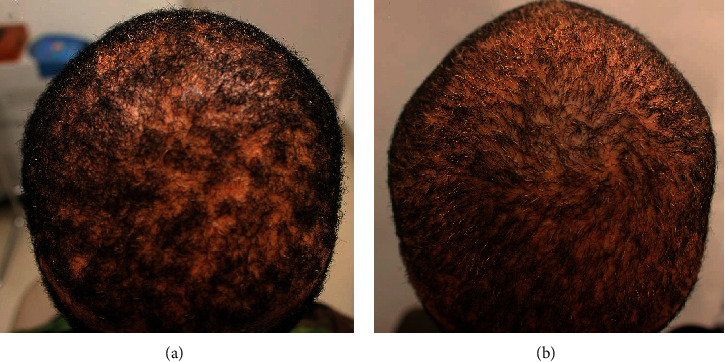
Patient 3: a 35-year-old African American man. Crown before (a) and after 3 months of treatment (b).

A 35-year-old African American male patient with hair loss used minoxidil and finasteride for 12 years with minimal benefits (Figure 6(a)). He applied GASHEE lotion to his hair and entire scalp twice a day. After 4 months of using the product, he reported decreased scarring and inflammation of his scalp and increased hair density in areas where he applied the lotion formulation (Figures 6(b)). He reported no adverse effects.

### 2.5. Case 4

**Figure 7:**
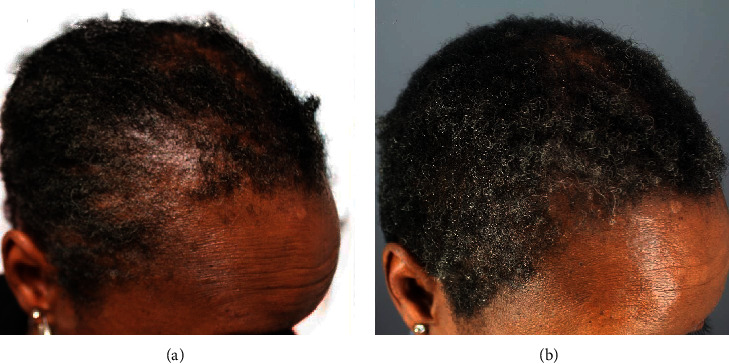
Patient 4: a 50-year-old African American woman diagnosed with central centrifugal cicatricial alopecia in the vertex and midscalp and traction alopecia in the temple, hairline, and nape areas. Right temple before (a) and after (b) 3 months of lotion use.

**Figure 8:**
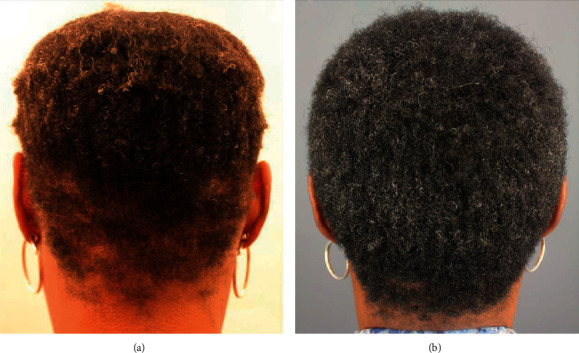
Patient 4: back of the head before (a) and after (b) 3 months of lotion use.

**Figure 9:**
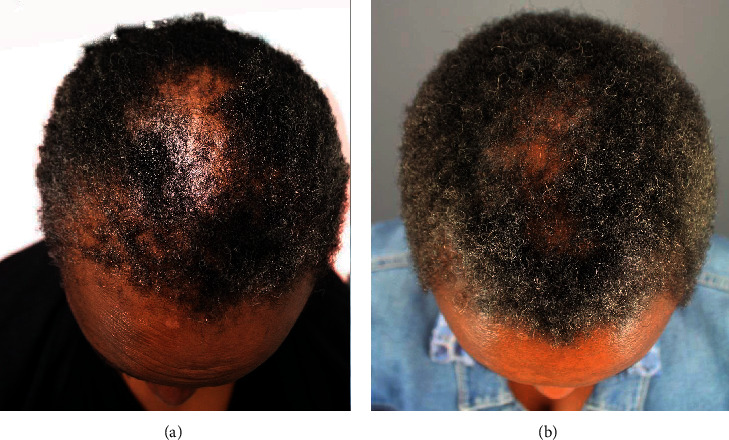
Patient 4: top of the head before (a) and after (b) 3 months of lotion use.

A 50-year-old African American woman had a history of hair loss in her hairline, temple edges, and the nape area, all consistent with a clinical diagnosis of traction alopecia (Figures 7(a) and 8(a)). Similarly, she had a biopsy-proven diagnosis of central centrifugal cicatricial alopecia involving the vertex (Figure 9(a)). She had used topical minoxidil for over 1 year on all areas of hair loss, as well as intralesional steroid injections, albeit with minimal benefits. After discontinuing all treatments, she began applying the GASHEE lotion twice a day. At 3 months, she reported significant improvement of hair fullness in the hairline, temples, and her nape areas of traction alopecia (Figures 7(b) and 8(b)). She equally observed some improvements in the vertex, with the exception of shiny scarred areas which were devoid of hair follicles (Figure 9(b)). She reported no adverse effects.

### 2.6. Case 5

**Figure 10:**
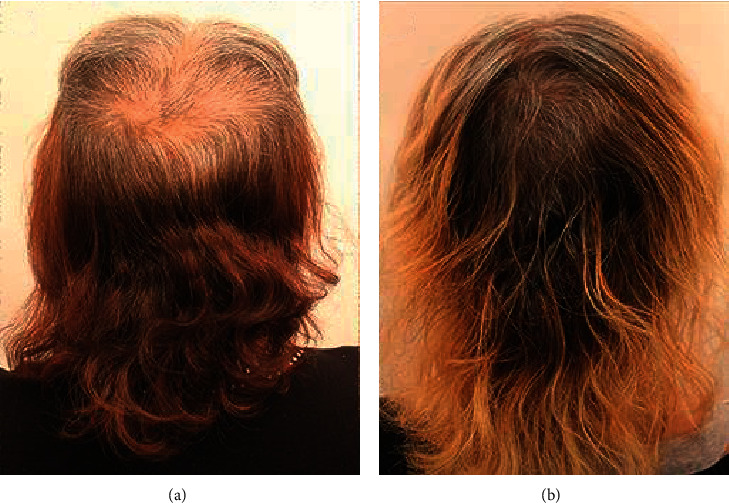
Patient 5: a 56-year-old, postmenopausal, Hispanic woman with marked thinning in the crown area, global loss of volume, and inability to grow hair long. Crown and back of head before (a) and after (b) 9 months of treatment.

A 56-year-old, postmenopausal, Hispanic woman completed chemotherapy for breast cancer and developed hair loss. When it regrew, she had generalized thinness with marked appearance in the crown, top, and frontal areas. She similarly reported a global loss of volume and inability to grow her hair long. Treatment with minoxidil for over 1 year showed no improvement. Within 6 weeks of initiating GASHEE lotion application, she noticed improvement in her global hair volume. Improvement was sustained through 18 months of documentation. She reported no adverse effects. Figures 10(a) and 10(b) depict the crown before and after 9 months of treatment.

## 3. Discussion

### 3.1. Dr. UGro Gashee®

Dr. UGro Gashee® (FineTouch Laboratories: Manhattan Beach, CA) is a novel, proprietary topical formulation consisting of over 12 different botanicals, vitamins, and cosmeceuticals, available as a liquid lotion or pomade, containing a similar amount of phytoactives. Each of its components has been incorporated to potentially apply a multifaceted approach to the treatment of AGA (Table 1). Similarly, the formulation contains methylsulfomethane, designed to aid penetration of the active topical ingredients [32]. Since it is used as a topical formulation, it minimizes adverse or toxic effects that are sometimes experienced after the ingestion of botanical extracts. The ingredients are derived by either cold pressing or using natural solvents, thus keeping in alignment with a holistic approach to cosmeceuticals and health care. In addition, the preparation of many topical botanical solutions involves the use of high temperatures to attain a desired texture or achieve maximum dissolution and emulsification. However, such an approach may destabilize or deactivate heat-sensitive phytochemicals, thus defeating the purpose of their use. Using ingredients obtained by implementing methods based on close-to-ambient temperatures and a formulation process that avoids heat, the biochemical or biological activity of active components is optimally retained.

Furthermore, GASHEE formulation favors using whole extracts as opposed to compounds isolated from plants. There is evidence suggesting that, at comparable doses and concentrations, industrially isolated compounds do not have as much activity as the unrefined plant due to the absence of interacting substances present in the extract [52].

Finally, one issue that has made many topical botanicals commercially nonviable is the inability to preserve them such that their shelf life is optimal without resorting to harsh chemical preservatives. GASHEE overcomes this challenge owing to its heat-free proprietary formulation process and blend of ingredients, as well as the naturally derived sustainable preservatives it contains. All of these factors are aimed at the increasing number of people who are reluctant to consume pharmaceuticals and oral medications and prefer topical natural solutions that are safer.

### 3.2. Dr. UGro Gashee® Ingredients and the Hair Follicle Cycle

Human hair follicles are in a constant state of cycling throughout their biological life. The cycle (Figure 1) consists of a growth phase (anagen) during which the follicle elongates and is at its thickest in caliber; a degeneration phase (catagen) which marks the end of anagen and is characterized by apoptotic changes involving the sections that extend from and include the hair bulb where DPCs reside to the bulge area where the hair stem cells live; and a resting phase (telogen) during which follicle miniaturizes and may shed (exogen). Resumption of anagen is marked by regeneration of the hair bulb and the hair follicle sections leading up to the follicle bulge area.

Several genes encode proteins that act as activators or inhibitors in the three stages of the hair follicle cycle (Figure 1). Understanding the mechanism underlying the influence of various components of the GASHEE formulation on many of these proteins will elucidate how it can be of benefit against hair loss. For example, one of the primary active constituents of green tea, epigallocatechin, is a DHT inhibitor, which prevents insulin-like growth factor- (IGF-) 1 levels from being depressed. IGF-1 prevents hair follicles from transitioning from anagen to catagen while encouraging the telogen to anagen transition [47]. *Eclipta alba* extract includes substances that can downregulate TGF-1*β*, a cytokine that encourages the hair follicle to transfer to the catagen phase, although the exact regulation pathway by which this occurs remains unknown [50]. Fenugreek extract can counteract the effects of proinflammatory cytokines, such as IL-6 and tumor necrosis factor- (TNF-) *α*, thus positively intervening in anagen-to-catagen and catagen-to-telogen stages of the hair cycle [53]. An abnormally functioning vitamin D receptor and inadequate vitamin D3 levels are known to disrupt the normal hair follicle cycle [51, 54]. Extracts of Gotu kola have been shown to be angiogenic. The angiogenesis marker, vascular endothelial growth factor, can mitigate androgen-induced apoptosis through the phosphoinositide-3-kinase-protein kinase B (Akt) pathway, which could affect the progression of anagen to catagen [55, 56].

### 3.3. Main Dr. UGro Gashee® Ingredients in Detail

#### 3.3.1. Gotu Kola

Gotu kola (*Centella asiatica* (*C. asiatica*)) is an herb used for thousands of years in the Ayurvedic Indian tradition, the activities of which have been attributed to several saponin moieties [57, 58]. Extracts of this herb have demonstrated antioxidant activity, as evidenced by several murine studies. For example, administration of Gotu extracts resulted in a significant oxidative defense in an Alzheimer's disease model [59]. In a neuroprotective model, *C. asiatica* mitigated the neurotoxic effects of 3-nitropropionic acid on oxidative stress in the cytosol and mitochondria and prevented the significant depletion of glutathione, total thiols, and other enzymic antioxidant systems [59, 60]. Finally, a significant decrease in malondialdehyde and an increase in glutathione and catalase levels were observed in male Wistar rats treated with 200 and 300 mg/kg of *C. asiatica* [33]. Notably, *C. asiatica* extracts were demonstrated to activate the hair-inductive capacity in cultured human DPCs [61].

#### 3.3.2. Green Tea Extract

Green tea extract is made from leaves of the plant, *Camellia sinensis* L. (Theaceae), in which enzymatic oxidation has been minimized. It is one of the most extensively studied botanicals due to its antioxidant, metal-chelating, anticarcinogenic, anti- and proapoptotic, and anti-inflammatory properties, originating from constituent catechins, 50–80% of which consist of epigallocatechin gallate (EGCG) [62]. An early study used BALB/black mice devoid of hair on the head, neck, and dorsal areas and randomly assigned them to an experimental group treated with 50% fraction of polyphenol extract from dehydrated green tea in their drinking water for six months and a control group given plain water. The authors showed that one-third of the mice in the experimental group had significant hair regrowth (*p*=0.014) compared to that in the control group [63]. Similarly, further investigation revealed that EGCG promotes hair growth in an *ex vivo* culture of hair follicles and the proliferation of cultured DPCs [64]. The authors suggested that growth stimulation of DPCs by EGCG *in vitro* was mediated by the upregulation of phosphorylated extracellular signal-regulated kinases and Akt, which are important for regulating cell growth, proliferation, survival, mobility, and invasion, as well as by the increased B-cell lymphoma 2/bcl-2-like protein 4 ratio, functioning like a rheostat to determine cell susceptibility to apoptosis. Notably, similar results were also obtained *in vivo* in dermal papillae of human scalps [64].

Finally, researchers used a micro-RNA (miRNA) microarray to identify miRNA expression levels in DPCs and determine the influence of this expression on the protective effects of EGCG against DHT-induced cell death, growth arrest, intracellular reactive oxygen species (ROS) levels, and senescence [65]. The experiments demonstrated that EGCG does protect against the effects of DHT by altering the miRNA expression profile in human DPCs, attenuating DHT-mediated cell death and growth arrest and decreasing intracellular ROS levels and senescence.

#### 3.3.3. Fenugreek

Fenugreek (*Trigonella foenum-graecum*) is a traditional medicinal herb commonly used in India, China, Thailand, and South-East Asian countries [66]. Bioactive molecules identified in extracts include saponins, flavonoids, coumarins, and alkaloids, targeting several molecules involved in inflammation and cancer cell proliferation, invasion, migration, angiogenesis, and metastasis [66, 67].

The antioxidative and anti-inflammatory properties of fenugreek have been noted in several studies [68–73]. It demonstrated a significant acute anti-inflammatory activity relative to water (control) and sodium diclofenac (standard) in mice with carrageenan-induced rat paw edema [68]. Diosgenin, the major steroidal sapogenin in the fenugreek seed, has demonstrated various anti-inflammatory functions, such as reducing the production of several inflammatory mediators, including nitric oxidase, IL-1, and IL-6, in murine macrophages [69, 70]. Similarly, it has been shown to inhibit superoxide generation in bone marrow-activated mouse neutrophils along with blocking cyclic adenosine monophosphate, protein kinase A, cytosolic phospholipase A2, p21-activated kinase, Akt, and mitogen-activated protein kinase (MAPK) signaling pathways [71]. This eventually leads to a reduction in the adhesive capacity of vascular smooth muscle cells (VSMCs) and TNF-*α*-mediated induction of intercellular adhesion molecule 1 and vascular cell adhesion molecule 1 in VSMCs by inhibiting the MAPK/Akt/nuclear factor-*κ*B signaling pathway and ROS production [72]. Additionally, it has been demonstrated to alleviate oxidative stress and inflammatory and apoptotic markers induced by monocrotaline [73].

In conclusion, the results reported here in five patients with hair loss show that meaningful improvement with a high safety profile was attainable using a topical botanical formulation that focuses on multiple targets of pathophysiologic hair-loss pathways. This formulation may present an alternative for people who wish to avoid the use of pharmaceuticals either due to concerns of adverse effects or suboptimal efficacy. A large randomized, double-blind, placebo-controlled study would be necessary to define the safety and efficacy of this formulation.

## Figures and Tables

**Table 1:** Components of Dr. UGro Gashee®.

Generic name	INCI name	Properties
Gotu kola	*Centella asiatica*	Activates hair-inductive capacity in three-dimensional spheroid cultured dermal papilla cells [33]
*Aloe barbadensis* extract	*Aloe barbadensis* extract	Immunomodulatory and anti-inflammatory properties [34]
Red (Asian) ginseng extract	Panax root ginseng extract	May reduce hair loss despite the presence of DKK-1, a strong catagen inducer via apoptosis [35]
Polygonum fo-ti extract	Polygonum multiflorum root extract	Increases the proliferation of dermal papillary cells [36]
Cysteine/N-acetyl-cysteine	Cysteine/N-acetyl cysteine	Androgen-inducible TGF-*β*1 promotes androgenetic alopecia; however, it is significantly suppressed by the ROS scavenger, N-acetyl cysteine [37]
Turmeric	*Curcuma longa*	Scavenger of reactive oxygen species, anti-inflammatory agent, and immunomodulator [38, 39]; anti-androgen [40] inhibits androgenic induction of TGF-*β*, which induces catagen and inhibits hair growth and perifollicular fibrosis [41–43]
Horsetail extract	*Equisetum arvense* extract	5*α*-reductase inhibitor [44]
Fenugreek oil	*Trigonella foenum-graecum* oil	Anti-inflammatory, antioxidant, antifungal, and antibacterial properties [45]
Tall oil fatty acids	Tall oil fatty acid	*β*-Sitosterol component may be an effective 5-alpha reductase inhibitor [46]
Green tea extract with 95% EGCG	*Camellia sinensis* leaf extract	Phytoconstituent epigallocatechin 3-gallate is a 5-*α* reductase inhibitor [47]
Saw palmetto	*Serenoa serrulata*	Inhibits both isoforms of 5-*α* reductase, preventing conversion of testosterone to its active form, DHT, and also promotes hair follicle vascularization [48, 49]
*Eclipta alba* oil	*Eclipta alba* (bhringraj oil)	Stimulates follicular keratinocyte proliferation and delays terminal differentiation by downregulating TGF-*β*1 expression [50]
Vitamin D3	Vitamin D3	Animal studies show that vitamin D3 analogs stimulate hair regrowth [51]

DHT: 5-dihydrotestosterone; DKK-1: Dickkopf WNT signaling pathway inhibitor 1; EGCG: epigallocatechin gallate; IGF-1: insulin growth factor 1; INCI: International Nomenclature of Cosmetic Ingredients; ROS: reactive oxygen species; TGF: transforming growth factor.

## Data Availability

In addition to photographic evidence showing interval changes that appear in the manuscript, complete medical records of the presented cases are stored in the treating clinic, in line with the standard of care.
